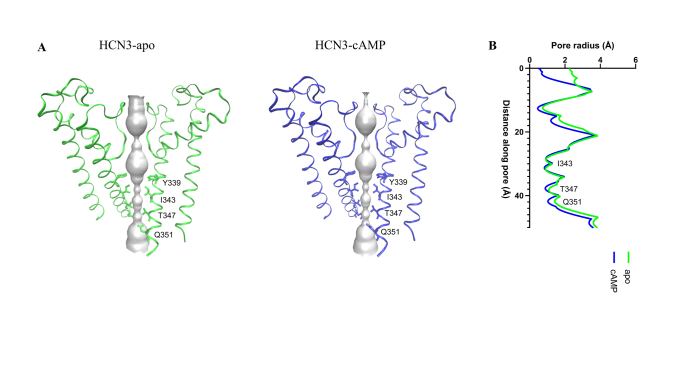# Cryo-EM structure of human HCN3 channel and its regulation by cAMP

**DOI:** 10.1016/j.jbc.2024.107288

**Published:** 2024-04-16

**Authors:** Bo Yu, Qiuyuan Lu, Jian Li, Xinyu Cheng, Han Hu, Yuanshuo Li, Tong Che, Yaoguang Hua, Haihai Jiang, Yuting Zhang, Cuiling Xian, Tingting Yang, Ying Fu, Yixiang Chen, Weiwei Nan, Peter J. McCormick, Bing Xiong, Jingjing Duan, Bo Zeng, Yanyan Li, Yang Fu, Jin Zhang

**Affiliations:** 1 The MOE Basic Research and Innovation Center for the Targeted Therapeutics of Solid Tumors, School of Basic Medical Sciences, Jiangxi Medical College, Nanchang University, Nanchang, China; 2 The Second Affiliated Hospital, Jiangxi Medical College, Nanchang University, Nanchang, China; 3 School of Medicine, Southern University of Science and Technology, Shenzhen, Guangdong, China; 4 College of Pharmacy, Gannan Medical University, Ganzhou, China; 5 Shenzhen Crystalo Biopharmaceutical Co, Ltd, Shenzhen, Guangdong, China; 6 William Harvey Research Institute, Bart’s and The London School of Medicine and Dentistry, Queen Mary University of London, London, UK; 7 Department of Medicinal Chemistry, Shanghai Institute of Materia Medica, Chinese Academy of Sciences, Shanghai, China; 8 Human Aging Research Institute (HARI), School of Life Sciences, Nanchang University, Nanchang, Jiangxi, China; 9 Key Laboratory of Medical Electrophysiology, Ministry of Education & Medical Electrophysiological Key Laboratory of Sichuan Province, Institute of Cardiovascular Research, Southwest Medical University, Luzhou, Sichuan, China; 10 Department of Chemical Biology, School of Life Southern University of Science and Technology, Southern University of Science and Technology, Shenzhen, Guangdong, China; 11 Institute for Biological Electron Microscopy, Southern University of Science and Technology, Shenzhen, Guangdong, China

**Keywords:** HCN3 channel, cryo-EM, Regulation, cAMP, CHS

## Abstract

HCN channels are important for regulating heart rhythm and nerve activity and have been studied as potential drug targets for treating depression, arrhythmia, nerve pain, and epilepsy. Despite possessing unique pharmacological properties, HCN channels share common characteristics in that they are activated by hyperpolarization and modulated by cAMP and other membrane lipids. However, the mechanisms of how these ligands bind and modulate HCN channels are unclear. In this study, we solved structures of full-length human HCN3 using cryo-EM and captured two different states, including a state without any ligand bound and a state with cAMP bound. Our structures reveal the novel binding sites for cholesteryl hemisuccinate in apo state and show how cholesteryl hemisuccinate and cAMP binding cause conformational changes in different states. These findings explain how these small modulators are sensed in mammals at the molecular level. The results of our study could help to design more potent and specific compounds to influence HCN channel activity and offer new therapeutic possibilities for diseases that lack effective treatment.

Hyperpolarization-Activated Cyclic Nucleotide-gated (HCN) channels represent a class of unique voltage-gated cation channel that are activated by membrane hyperpolarization and modulated by cyclic nucleotide and membrane lipids. The four mammalian subtypes, HCN1, HCN2, HCN3, and HCN4 share about 60% sequence identity with each other and 25% sequence identity with their closest relatives-the cyclic nucleotide-gated channels (1). HCNs are wildly expressed in heart and the nervous system (2). HCN channels play a major role in neuronal excitability and cardiac pacemaker activity and have emerged as attractive targets for the treatment of various heart and central nervous system disorders, including heart failure, depression, neuropathic pain, and epilepsy. Ivabradine is the first and only HCN inhibitor, currently approved for the treatment of chronic stable angina pectoris (3). However, ivabradine and its currently available analogs are not selective for HCN channels and have considerably modest IC50 values in the low micromolar range (4), which might explain its various adverse effects, including irregular heartbeat, blurred vision, and headache disorder (5). The clinical translation of inhibitors targeting these channel subtypes has remained largely unsuccessful. To understand the interaction of HCN with small molecule compounds and to facilitate the development of more specific and potent inhibitors for clinical use, structural insight into drug and inhibitor binding to HCN channels is urgently needed.

Previous studies have reported that the HCN3 channel is not only expressed in the olfactory bulb, hypothalamic nuclei and retinal cone in the nervous system (6, 7, 8, 9), but is also abundant in heart muscle,kidney and other tissues (10, 11, 12, 13). Of note, in contrast to HCN1, HCN2, and HCN4, HCN3 is highly expressed in thalamic intergeniculate leaflet neurons, a nucleus which regulates circadian rhythms, and is responsible for pacemaker current (Ih), suggesting that HCN3 plays a critical role in sleep and circadian rhythms by regulation of neuronal excitability and oscillatory activity in intergeniculate leaflet neurons (14). While deletion of HCN3 demonstrated no obvious organ specific phenotype, behavioral studies suggest that HCN3-deficient mice are impaired in processing contextual information (12). Furthermore, overexpression of HCN3 is implicated in the development and progression of triple-negative breast cancer (15). HCN3 also play a protective role in the kidney serving as a novel mitochondrial K^+^ channel that is coupled to the respiratory chain and ATP synthesis (16). As a result, the treatment of a multitude of diseases might benefit from inhibition of HCN3.

Due to the important role of HCN channels in the regulation of excitability in neurons and the heart, understanding how lipids regulate HCN channels has been an intense area of research for many years. Several membrane lipids, including cholesterol, phosphoinositides, and sphingolipids, have recently been shown to regulate HCN channel function (17, 18, 19, 20, 21, 22, 23). The channel function depends largely on cholesterol, which can regulate various ion channels by directly interacting with them or by altering the membrane’s physical properties (24, 25, 26). In most cases, cholesterol has been shown to decrease the activity of channels, while other lipids such as phosphoinositides, including phosphatidylinositol 4,5-bisphosphate do the opposite, increasing the channel activity (27, 28). Even though lipid regulation of ion channels is widely studied, and more local structural of HCNs are being resolved (29, 30), while there is no available structure highlighting direct HCN–lipid interactions.

The recent cryo-EM structures of HCN1 (31) and HCN4 (32) unveiled a tetrameric topology, with an intracellular N-terminal domain, including the HCN domain, six transmembrane domains (TMDs), and C-terminal domain, including C-linkers and cyclic nucleotide-binding domain (CNBD), which contains the binding site of cAMP. Whereas the TMDs are nondomain-swapped and form a nonselective cation channel mainly permeable to Na^+^ and K^+^. The pharmacology of HCN channel subtypes is also divergent with the sole common feature being activation by hyperpolarization and allosteric modulation by cAMP and other membrane lipids. However, the regulatory mechanism of human HCN3 channel modulation by various effectors remains poorly understood. To understand the mechanism of ligand and lipid sensing by HCN channels, we determined two cryo-EM structures of HCN3:ligand-free and cAMP-bound structures. Our structural and functional studies identified distinct binding sites of cAMP, revealing the underlying structural basis for the conformational changes in the CNBD induced by cAMP modulators. Moreover, our current work contributes to a better understanding on the molecular details of HCN subfamily members as well as providing new insights into design subtype-specific inhibitors.

## Results

### Overall structure of HCN3 channel

**Supporting Figure S1:**
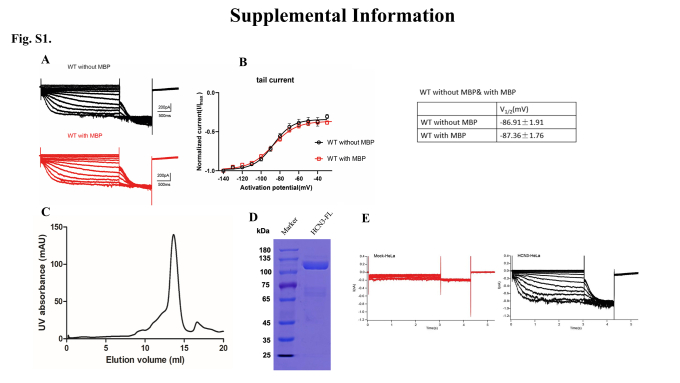


To gain insight into the structure of HCN3 channel, the full-length wild-type human HCN3, with its N terminus fused to maltose-binding protein (MBP), was expressed using the BacMam expression system (Experimental procedures).The MBP tag was retained during subsequent structural determination, as its cleavage significantly reduced protein yield and stability. As expected, with or without the MBP tag, HCN3 was activated by membrane hyperpolarization when expressed in HEK cells (Fig. S1, *A*, *B*, and *E*).The protein was solubilized and purified in the detergent lauryl maltose neopentyl glycol with cholesteryl hemisuccinate (LMNG/CHS), followed by exchange into digitonin for single-particle cryo-EM studies. Purified proteins were characterized as stable and monodispersed (Fig. S1, *C* and *D*).

**Figure 1:**
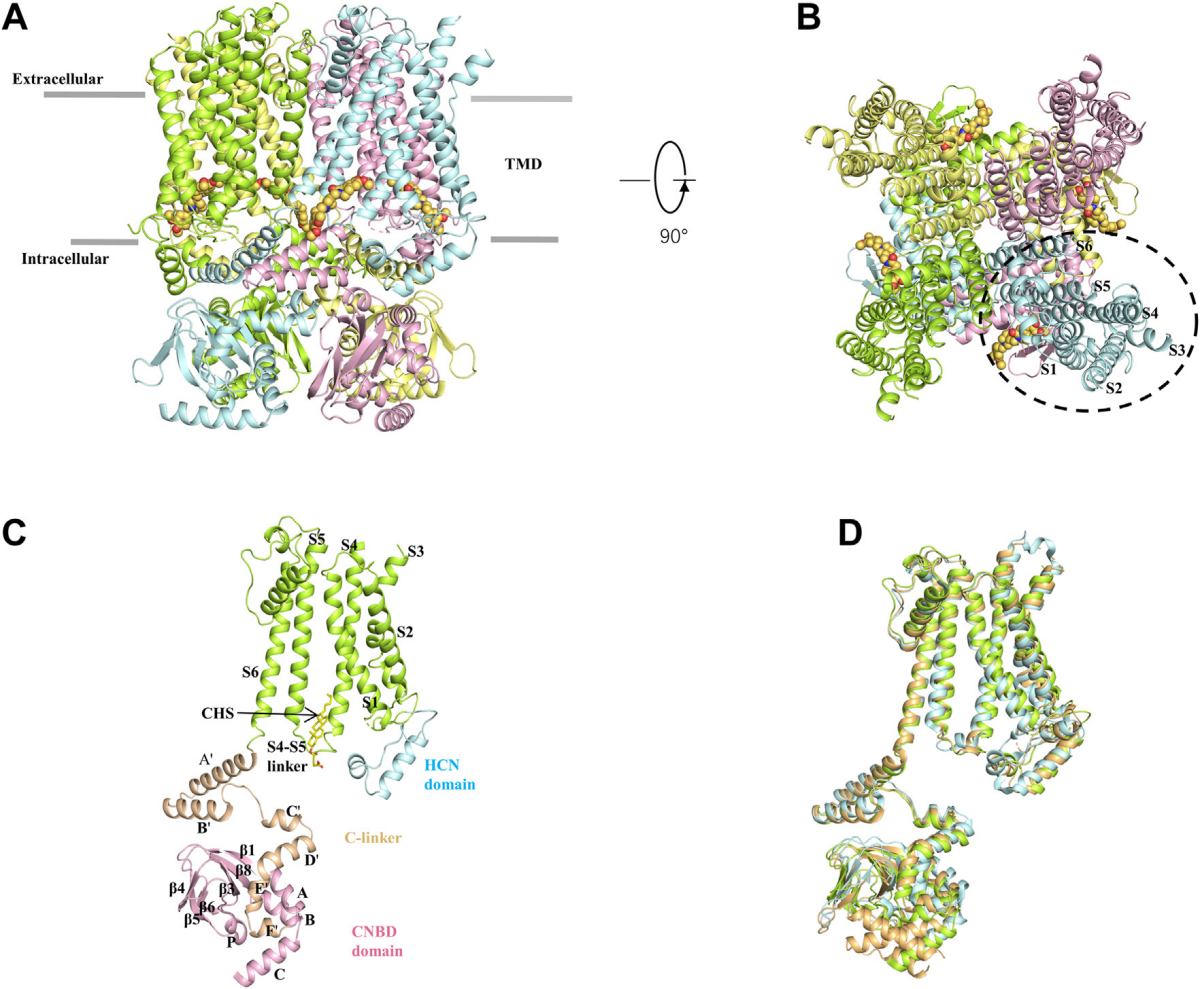
**Architecture of the human HCN3 channel and its comparison with HCN1 and HCN4.***A* and *B*, the structure of the HCN3 channel tetramer in the apo state, viewed parallel to the membrane (*A*) or from the extracellular side (*B*). Each subunit is depicted in a different color. The *gray bars* denote the approximate boundaries of the membrane bilayer. TMD refers to the transmembrane domain and CNBD to the cyclic nucleotide-binding domain. *C*, monomer of human HCN3 with indicated structural elements. The cholesteryl hemisuccinate (CHS) at the S4-S5 linker is shown due to the high resolution. *D*, comparison of HCN3 with HCN1 (PBD ID: 5U6O) and HCN4 (PBD ID: 7NP3). For clarity, only one subunit in the tetramer is fully displayed. The HCN3 structure is colored *green*, HCN1 is colored *orange*, and HCN4 is *cyan*. HCN, Hyperpolarization-Activated Cyclic Nucleotide-gated.

**Supporting Figure S2:**
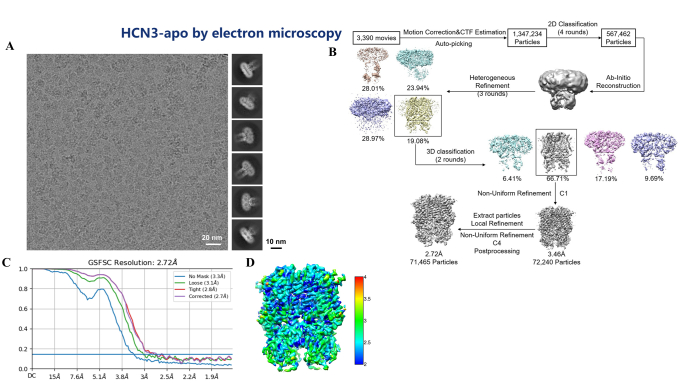


**Supporting Figure S3:**
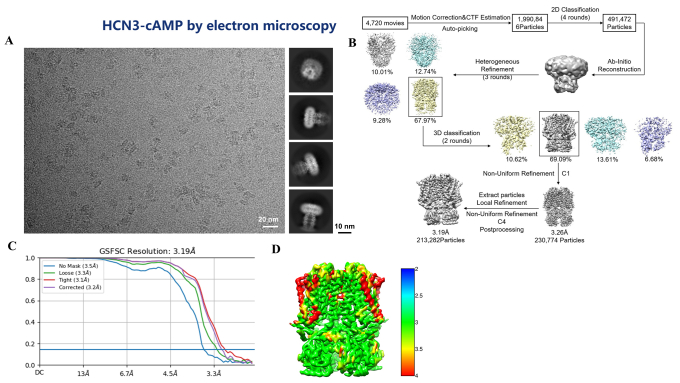


**Supporting Figure S4:**
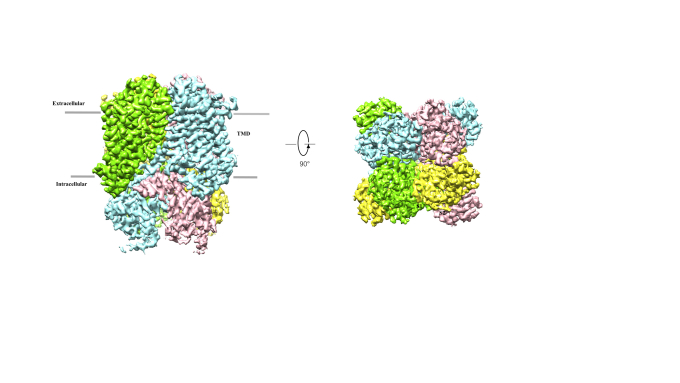


Two representative structures were determined, including the ligand-free (apo-HCN3) and cAMP-bound (cAMP-HCN3) structures, at an overall resolution of 2.7 Å and 3.1 Å, respectively (Figs. S2 and S3). There was a good correlation between the final models and the density maps. The last 231 C-terminal amino acid residues of HCN3 (residues 544–774) were found to be too disordered to build a model and were predicted to lack secondary structure (Figs. 1, *A*–*C* and S4)), indicating their flexibility.

Overall, the structure of HCN3 closely resembles that of previously solved HCN1 and HCN4 structures (Fig. 1*D*), characterized by a similar positioning of the six-transmembrane (S1–S6) bundle. HCN3 is observed to form a 4-fold symmetric tetramer, with each protomer comprising three structural layers: the HCN homology domain located at the N terminals in the cytoplasmic region, the nonswapped TMD, and the C-terminal domain containing the C-linker (helices A′ to F′) and CNBD (six-strands (β1–β6) and four helices (A–C and P) arranged in a domain-swapped manner. The transmembrane bundles of HCN3, consisting of six α-helices (S1–S6) and a loop domain situated between the S5 and S6 helices that forms the ion selectivity filter, are highly similar to those observed in other HCN channel structures. Despite the amino acid sequence of the S4 to S5 linker being identical among HCN channels, the S4 to S5 linker of HCN3 adopts a loop conformation, in contrast to HCN1 and HCN4, suggesting a more flexible modulation of the movement of the “voltage sensor-like” S1 to S4 domains to the channel gate.

### CHS binding site

**Figure 2:**
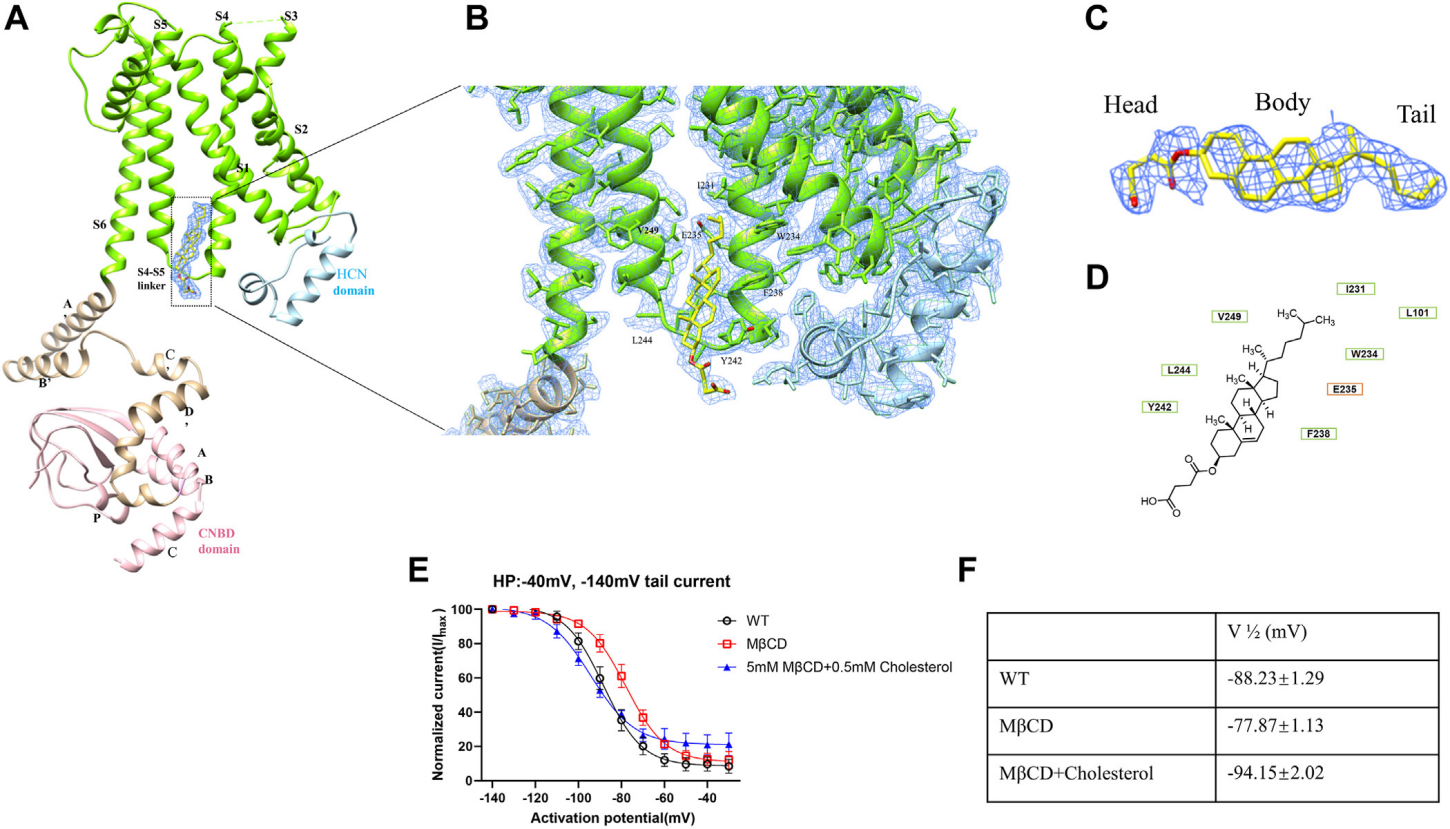
**CHS binding site and its effect on HCN3 channel.***A*, *cartoon representation* of one subunit of HCN3 channel with individual domains colored. *B*, CHS-binding pocket. The discernible nonprotein density observed within the intracellular domain of HCN3, created by the terminal regions of S4, S5, and the S4-S5 linker, tightly accommodates CHS (colored as *yellow*). *C*, the “head,” “body,” and “tail” of CHS are referred to as the hemisuccinate group, steroid ring, and alkane group, respectively. *D*, chemical structure of CHS and its interaction with surrounding amino acids. *E*, steady-state activation curves of WT HCN3, MβCD-treated HCN3, and reintroduced cholesterol-treated HCN3 currents obtained by whole-cell voltage-clamp recordings showed that HCN3 channel is inhibited by cholesterol. Data points are mean ± SEM of three to five cells. *F*, the voltage of half-maximal activation (V1/2) determined by Boltzmann fit to the mean of steady-state activation curve from Figure 2*E*. CHS, cholesteryl hemisuccinate; CNBD, cyclic nucleotide-binding domain; HCN, Hyperpolarization-Activated Cyclic Nucleotide-gated; MβCD, methyl-β-cyclodextrin.

**Supporting Figure S5:**
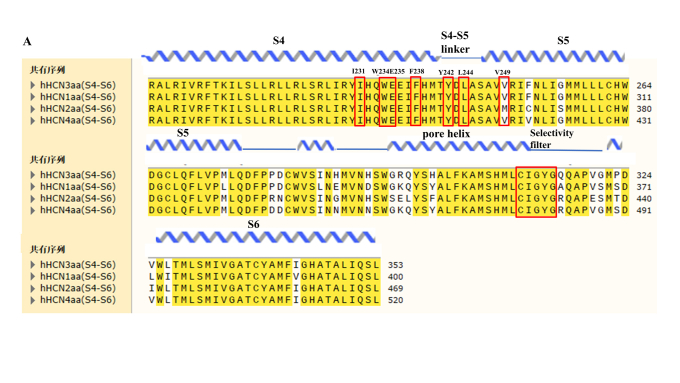


An intriguing finding in our study is the presence of a prominent density located at the interface of the membrane and cytosol in the apo channel. This density has a shape that matches well with that of CHS, a water-soluble cholesterol analog employed during the protein purification process using LMNG as a detergent (Fig. 2, *A*–*C*).The molecular structure of CHS includes four steroid rings fused in a planar configuration and a hydrophobic tail forming together a highly hydrophobic structure. The hemisuccinate group, steroid ring, and alkane group are referred to as the “head”, “body”, and “tail”, respectively. The hydrophobic interface at the intracellular part of HCN3 created by the ends of S4, S5, and S4-S5 linker, tightly accommodates CHS (Fig. 2, *A*–*C*). The residues I231, W234, E235, and F238 in S4, Y242, and L244 in the S4-S5 linker, and V249 in S5 are involved in CHS binding (Fig. 2*B*). Among these, Y242 plays a major role in interacting with the steroid hydrophobic rings of CHS. The residues involved in the interaction with CHS are highly conserved in HCN channels, indicating that lipids play a crucial role in the structural stabilization of HCN channels (Fig. S5). While this may represent a shared characteristic among all HCN channel structures, it is noteworthy that in the density maps of HCN1 and HCN4, the density corresponding to cholesterol was not observed. This discrepancy could be attributed to variations in purification methods or differences in resolution.

**Supporting Figure S6:**
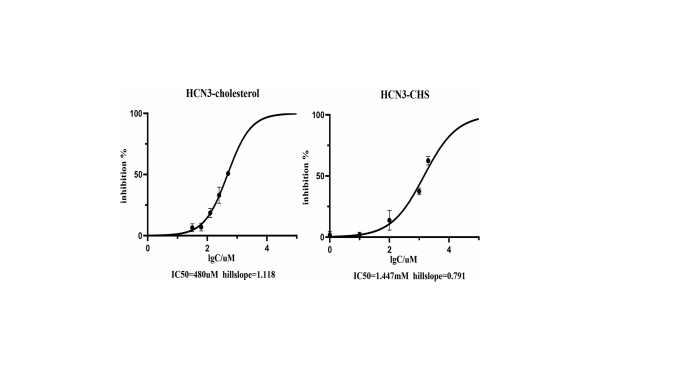


It is reported that the S4 helix and S4-S5 linker are key positions for gating. Hyperpolarization in the HCN channel creates an interfacial helix out of the C-terminal end of the S4 helix (33, 34). CHS seems to stabilize the HCN structures in the closed state. Similar observations have been made in the HCN4 structure, where it appears that the parallel arrangement of transmembrane helices is stabilized by lipids (32). In particular, the presumed lipid appears to make contact with the lower part of the S4 helix, where it connects to the S4-S5 linker. To verify the inhibitory effect of cholesterol or CHS, we used methyl-β-cyclodextrin (MβCD) to remove endogenous cholesterol from our experimental system and record the currents of HCN3 on HeLa cells by electrophysiology. In the context of steady-state activation curves, treatment with MβCD was found to result in a rightward shift of channel activation by a magnitude of 10 mV (Fig. 2, *E* and *F*). This shift implies that HCN3 is more readily activated in the absence of cholesterol and its derivatives. Importantly, our findings indicate that depletion of cholesterol *via* MβCD treatment led to a significant hyperpolarization of HCN3, thereby implying the existence of endogenous cholesterol which negatively modulates HCN3 function. Moreover, the reintroduction of cholesterol resulted in a leftward shift of the steady-state activation curve, leading to a pronounced reduction in the open probability of HCN3. These outcomes provide further support for the inhibitory effect of endogenous cholesterol on HCN3 activity. To supplement these observations, we tested the IC50 values of cholesterol and CHS, which were measured to be 480 μM and 1.45 mM, respectively (Fig. S6). Taken together, our results underscore the plausible involvement of endogenous cholesterol as a key regulator of HCN3 activity in cellular contexts (Fig. 2, *E* and *F*).

### Conformational changes induced by cAMP

The HCN3 channel is activated by hyperpolarization and allosterically modulated by cAMP and other membrane lipids. To investigate the conformational changes that HCN modulators induce, we performed cryo-EM analyses of full-length human HCN3 in physiological conditions which bind with cAMP.

**Figure 3:**
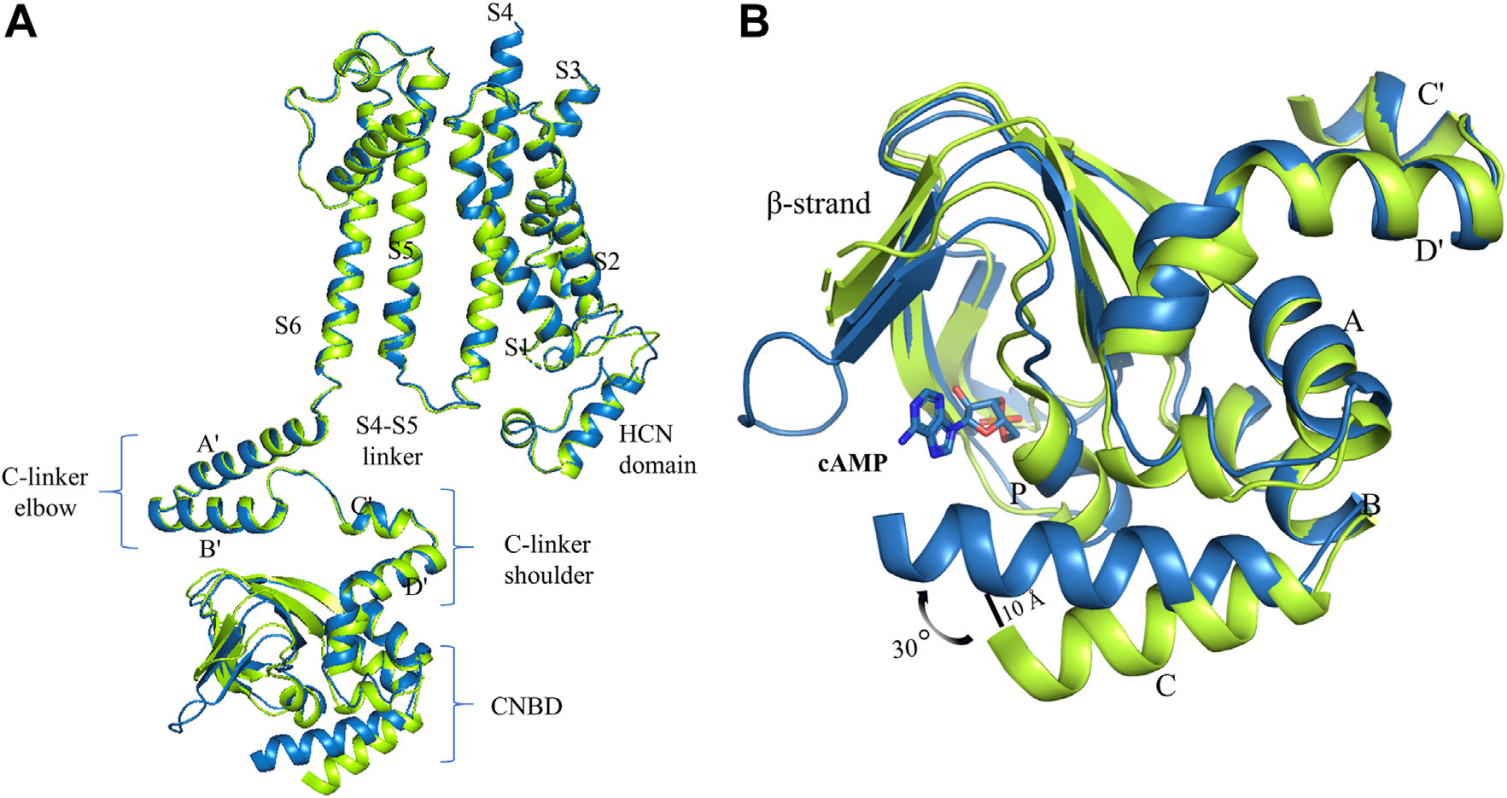
**Conformational changes upon cAMP binding.***A*, comparison of two HCN3 states, including ligand-free (PBD ID: 8INZ) and cAMP-bound (PBD ID: 8IO0) states, reveal high similarity (apo-*green*, cAMP-*blue*). *B*, superposition of CNBDs in the ligand-free (*green*) and cAMP-bound (*blue*) states based on the Cα atoms of β-strand and A, B, C, and P helices. The cAMP molecule is depicted in *sticks*. CNBD, cyclic nucleotide-binding domain; HCN, Hyperpolarization-Activated Cyclic Nucleotide-gated.

The overall structure of cAMP-HCN3 showed high similarity to the apo-HCN3 structure (RMSD = 0.529) (Fig. 3*A*). The TMDs of cAMP-bound HCN3 closely resemble those of apo-HCN3 conformation, and the structure of TMD of HCN3 in the presence of cAMP was similar to that in its absence, indicating that addition of cAMP alone is insufficient to open the channel. However, the CNBDs of the two were very different (Fig. 3*B*). The superimposition of a single subunit from the apo- and cAMP-bound structures shows that cAMP-binding causes rigid body movements of CNBD domains, resulting in a 10 Å closure of the cleft between C helix and β strand 4 (Fig. 3*B*). Secondly, the density of CHS was not observed in cAMP-bound structures, which is consistent with the idea that CHS represents an inactivating lipid. To investigate the significance of cholesterol in the structure and function of HCN3 in the absence of cholesterol and CHS, we attempted to purify HCN3 using cell lysate preincubated with MβCD to remove endogenous cholesterol. However, under these conditions, we were unable to obtain purified HCN3 protein without CHS, suggesting that endogenous cholesterol or CHS is essential for the stability of the HCN3 protein. These results emphasize the critical role of cholesterol in maintaining the structural and functional integrity of HCN3 protein.

### cAMP-binding pocket

**Figure 4:**
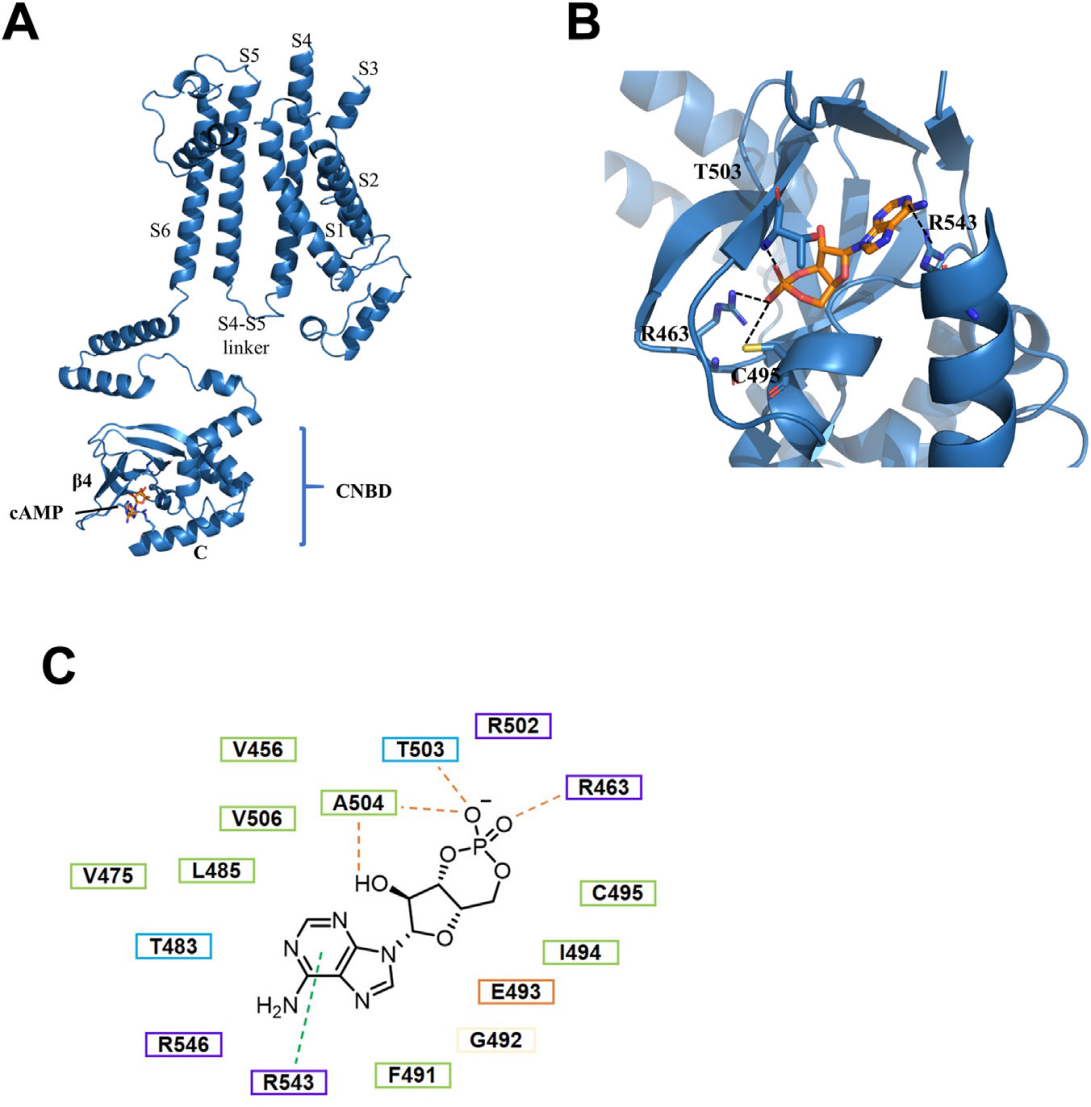
**cAMP-binding pockets.***A*, the cAMP-bound protomer of HCN3 is shown in *cartoon form*, with the binding pocket depicted at a cleft between the C helix, P helix, and β strand 4. The cAMP molecule is colored in *blue*. *B*, *close-up view* of the cAMP binding site of HCN3, highlighting the hydrogen bonds and salt bridges that stabilize the cAMP molecule. Four residues (R463, C495, T503, and R543) are depicted as *sticks* and labeled. *C*, chemical structure of cAMP and interacted residues. HCN, Hyperpolarization-Activated Cyclic Nucleotide-gated.

In the cAMP-HCN3 structure, we observe a strong density that bridges the C helix and β strand 4 at the site where cAMP is expected to bind. This density is well suited to accommodate the cAMP molecule. The cAMP is coordinated in a crevice formed between the alpha helices and the β roll. The cAMP-binding pocket is located at a cleft between the C helix, P helix, and β strand 4, which together form the CNBD of HCN3 (Fig. 4*A*). The cAMP-HCN3 structure reveals that the residue R543 on the C helix forms hydrogen bonds and cation–π interactions with the AMP of cAMP, primarily stabilizing the CNBD domain. The phosphate group of cAMP is pointed inward within the cAMP-binding cleft and is accommodated by residues R463, C495, and T503 (Fig. 4, *B* and *C*). Notably, the positively charged residue R543 on the C helix undergoes substantial shifts in the cAMP-HCN3 structure, with the entire domain moving toward the cAMP-binding pocket. Specifically, the C helix in the CNBD shifts 30 degrees (Fig. 3*B*). The overall geometry and composition of the cAMP-binding pocket are consistent with observations in crystallographic studies of the CNBD domain of HCN2 and cryo-EM studies of the cAMP-HCN1 structure and have been confirmed by extensive mutational studies (35, 36, 37, 38, 39).

## Discussion

Although HCN channels have been extensively studied, the molecular mechanism of regulation by lipids and small molecule inhibitors remains elusive. In this study, we report cryo-EM structures of human HCN3, which is one of the four members of HCN channels, in apo and complexes with cAMP. Our structures reveal novel binding pockets for CHS and cAMP in HCN3. These findings shed light on how cholesterol, which is a major lipid component of mammalian cell membranes, modulates HCN channel function (28, 40).

**Figure 5:**
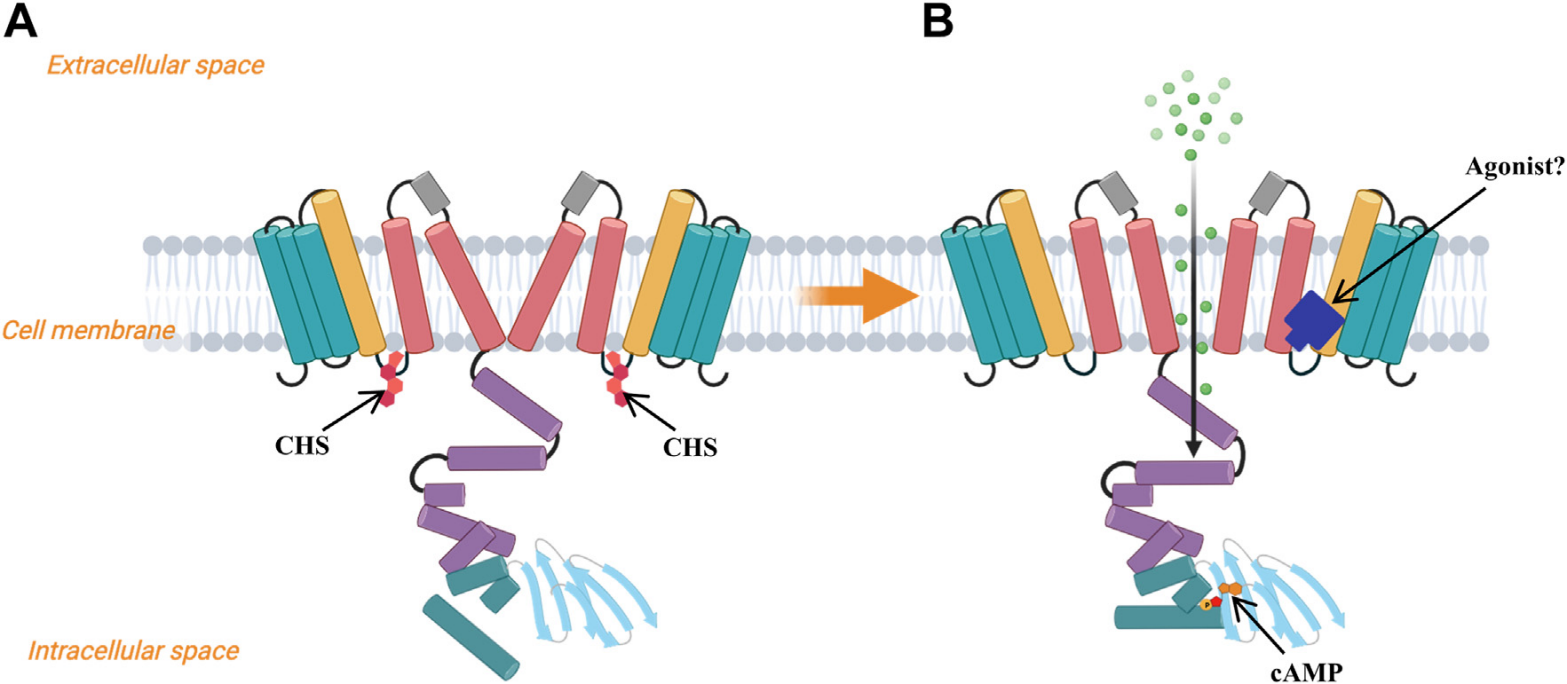
**Schematic model of HCN3 modulation by cholesterol/CHS and cAMP.***A*, in the absence of ligands or in the presence of inhibitory molecules, cholesterol/CHS binds to the S4-S5 linker and inhibits the opening of the channel. *B*, in the activation state induced by membrane hyperpolarization and possibly more potent agonists, the channel is *open*. HCN, Hyperpolarization-Activated Cyclic Nucleotide-gated; CHS, cholesteryl hemisuccinate.

We identified a novel binding site for cholesterol located at the hydrophobic interface created by the S4 and S4-S5 linker, which is a key position in the gating mechanism. It has been previously reported that the S4 helix undergoes a conformational change from a single helix in the depolarized state to two helices in the hyperpolarized state (33). Our findings suggest that CHS may stabilize the HCN3 structure in the closed pore state by interacting with the S4 and S4-S5 linker (Fig. 5*A*). Previous analysis of the effects of cholesterol on other HCN channels found differential effects (28). Our findings have interesting implications on what high cholesterol levels might mean for HCN3 activity. In contrast to the inhibitory role of cholesterol on channel activity, other lipids such as phosphatidylinositol 4,5-bisphosphate(PI(4,5)P_2_) is known to potentiate the activity of HCN 1 to 4 channels (41, 42, 43, 44, 45). These may imply that different binding sites in HCN3 mediate a diverse mechanism of HCN3 modulation by membrane lipids.

The apo-HCN3 and cAMP-HCN3 structures exhibit remarkable similarity. Similar to HCN1, cAMP alone is insufficient to open the channel. In the modulation of HCN3 channel activity, cAMP may serve as a sensor, initiating conformational changes upon activation. Binding of cholesterol or CHS to the interfacial cavity near the S4-S5 linker in the apo state obstructs the substantial conformational changes required for channel activation, resulting in a closed channel pore (Figs. 5*A*, and S7) (46, 47). This mechanism is akin to the endogenous phosphatidylinositol (PI) lipid in the transient receptor potential vanilloid 1 (TRPV1) channels, where PI lipid binds to the S4-S5 linker and stabilizes TRPV1 in the closed state (48). TRPV1 agonists replace PI lipid to induce channel opening. Therefore, we speculate that HCN may have a similar activation mechanism, where an agonist substitutes for cholesterol, leading to channel opening (Fig. 5*B*). However, in light of the absence of a structure depicting the open state of HCN bound to an agonist, the requirement for simultaneous displacement of CHS molecules and agonist binding to activate the channel remains uncertain. The future study is imperative to delineate the activation mechanism and identify the binding sites of potent agonists to HCN3.

In summary, our full-length human HCN3 structure enables new comparisons between the different subgroups of the HCN channels and advance the understanding how lipids regulate HCN channel activity. Given the therapeutic potential of HCN3 inhibits the treatment of triple-negative breast cancer and sleep disorders, the structure of HCN3 insights reveal critical interactions in a key state of the channel, which will allow medicinal chemists and computational biologists to design new potent and subtype-specific inhibitors.

## Experimental procedures

### Construct design, protein expression, and purification

A DNA sequence encoding the complete human HCN3 channel was synthesized and cloned into the pEGBacMam vector. To facilitate protein purification, a MBP tag was added to the N terminus of HCN3 *via* a linker containing the HRV 3C cleavage site. The full-length HCN3 protein was then expressed in HEK293S cells using the BacMam method. Briefly, a bacmid carrying the HCN3 construct was generated by transforming *Escherichia coli* DH10Bac cells with the HCN3 construct according to the manufacturer's instructions (Bac-to-Bac; Invitrogen). Baculo viruses were subsequently produced by transfecting *Spodoptera frugiperda* Sf9 cells with the bacmid using Cellfectin II (Invitrogen), and baculo viruses after two rounds of amplification were used for cell transduction. Suspension cultures of HEK293S cells were grown at 37 °C to a density of 3 × 10^6^ cells/ml and baculo viruses were added (10% v/v) to initiate the transduction. After 12 h, 10 mM sodium butyrate was supplemented, and the temperature was shifted to 30 °C. Cells were harvested at 72 h posttransduction.

Prior to solubilization, cells were resuspended for 30 min in a hypotonic lysis buffer containing 20 mM KCl, 0.5 mM MgCl_2_, 2 mM DTT, and 10 mM Tris pH 8.0 with EDTA-free protease inhibitor mixture. The lysate was then homogenized over 40 times and filled to a volume of 25 ml, followed by rolling at 4 °C for 2 h. The lysate was then centrifuged at 39,800*g* for 35 min to sediment the crude membranes, which were then homogenized and filled to a volume of 25 ml with lysis buffer, followed by the addition of detergent (LMNG:CHS = 5:1) at a concentration of 0.5% w/v. This was then rolled at 4 °C for 3 h, after which the solubilized membranes were clarified by centrifugation at 39,800*g* for 35 min. The supernatant was then applied to MBP beads and rolled at 4 °C overnight. The following day, the protein was washed with ten column volumes of wash buffer (0.1% digitonin, 300 mM KCl, 2 mM DTT, and 20 mM Tris pH 8.0) and eluted with four column volumes of elute buffer (0.1% digitonin, 300 mM KCl, 2 mM DTT, and 20 mM Tris pH 8.0, 40 mM maltose). The protein was then concentrated by Amicon Ultra centrifugal filter (molecular weight cut off 100 kDa) and injected onto a Superose 6 column (GE Healthcare), equilibrated with size exclusion chromatography buffer (0.1% digitonin, 300 mM KCl, 2 mM DTT, and 20 mM Tris pH 8.0). Peak fractions were pooled and concentrated to 8.8 mg/ml. All buffers contained protease inhibitors (2 mg/ml leupeptin, 1 mg/ml pepstatin, 50 mg/ml benzamidine, 10 mg/ml aprotinin, and 1 mM AEBSF hydrochloride, serine protease inhibitor). Additionally, 0.1 mM cAMP was supplemented to all buffers to purify the channel in the cAMP-bound state, and 5 mM cAMP was spiked into the protein sample prior to EM grid preparation.

### Cryo-EM sample preparation and data acquisition

A total of two different samples were reported in this study. For the apo structure sample, HCN3 protein was concentrated to 8.8 mg/ml. For the HCN3 sample with cAMP, 8.8 mg/ml HCN3 was incubated with cAMP at a final concentration of 5 mM on ice for 1 h. To prepare cryo-EM grids, 3 μl of samples were added to 400 Mesh R2/1Cu Quantifoil grids (glow discharged at 15 mA for 40 s with a glow discharge cleaning system). Grids were blotted with qualitative filter paper in a Vitrobot Mark IV (Thermo Fisher Scientific) at 4 °C and 100% humidity for 3 to 4 s using a blot force of −2 prior to plunging into liquid ethane. For cryo-EM data acquisition, grids were loaded on a Thermo Fisher Scientific 300 kV Transmission Electron Microscope Titan Krios equipped with Gatan K3 direct electron detector. Raw movies were collected using SerialEM in super-resolution mode. The data collection parameters for the different samples were summarized in Table 1 for details.

**Table 1:** Cryo-EM data collection parameters for the different samples

Samples	Number of micrographs	Total dose (e−/Å2)	Physical pixel size (Å)	Spherical aberration (mm)	Frame	Nominal defocus range (μm)
HCN3-apo	3390	50	0.842	2.7	32	−1 to −2
HCN3-cAMP	4720	40	0.668	2.7	32	−1 to −2

Abbreviation: HCN, Hyperpolarization-Activated Cyclic Nucleotide-gated.

### Cryo-EM data processing

For the different samples, super-resolution image stacks were gain-normalized and imported into cryoSPARC v3.3.2. After motion correction, electron-dose weighting, and contrast transfer function estimation, the initial particle was identified using the cryoSPARC auto picker. Particles were selected and cleaned through several rounds of 2D classification, which were then subjected to *ab initio* reconstruction in C1 using cryoSPARC. The resulting reconstructions were subsequently employed as models for heterogeneous refinement in cryoSPARC with all nonjunk particles. The particles that gave a reconstruction with channel features were then 3D classified globally and subjected to nonuniform refinement in C1 within the best class. To further enhance the resolution, the final particle sets were re-extracted with original box size and further applied for final nonuniform refinement and local refinement in C4 symmetry in cryoSPARC, resulting in a density map with overall resolution determined by gold standard Fourier shell correlation using the 0.143 criterion.

### Model building

The initial model of HCN3-apo was constructed based on the HCN1-apo cryo-EM structure (PDB: 5U6O) (31). *De novo* model building, guided by densities for bulky side chains and disulfide bonds, was conducted using the COOT (https://www2.mrc-lmb.cam.ac.uk/personal/pemsley/coot/) software (49). Subsequent cycles of model building in COOT and real-space refinement using real space refine against the full map in PHENIX (50) were performed to obtain the final refined atomic model, which was validated using the MolProbity program (51). The final HCN3-apo structure includes residues 50 to 151, 156 to 191, 210 to 476, and 484 to 543, whereas the cAMP-bound structure includes additional residues 477 to 483. The disordered N-terminal and C-terminal regions of HCN3 were not modeled for residues with poor density. Pore radius calculation was performed using the HOLE program (52), and structural figures were generated using the PyMol software (pymol.org).

### Electrophysiology and data analysis

Various constructs of the human HCN3 were cloned into the pcDNA3.1 vector for electrophysiological experiments. HeLa cells were transiently transfected using lipofection, and whole-cell patch-clamp recordings were obtained using the HEKA EPC10 amplifier and PATCHMASTER (https://www.heka.com/downloads/downloads_main.html#down_patchmaster) software, 12 h post-transfection. The extracellular solution contained 110 mM NaCl, 0.5 mM MgCl_2_, 1.8 mM CaCl_2_, 5 mM Hepes, 30 mM KCl, and pH was adjusted to 7.4 with NaOH, while the pipette solution contained 130 mM KCl, 10 mM NaCl, 0.5 mM MgCl_2_, 1 mM EGTA, 5 mM Hepes, and pH was adjusted to 7.4 with KOH. The recordings were performed at room temperature (22–25 °C), voltage-clamp mode, and filtered at 2 kHz. The sampling frequency was 50 kHz, and the series resistance was limited to 5 MΩ. To elicit channel currents, step pulses of 3 s duration ranging from −140 mV to −30 mV were applied, followed by a step to −140 mV lasting 1.5 s. The tail currents evoked by the second step were used to determine the voltage-dependent activation curves and calculate the midpoint of activation potential (V1/2). The IC50 of drugs was determined by recording currents at a pulse of −100 mV and analyzing current amplitudes at varying drug concentrations. The IC50 value was calculated using the Hill equation. To investigate the effect of cholesterol and CHS on HCN3 channel, endogenous cholesterol was removed by incubating cells with 5 mM MβCD for at least 60 min, and cholesterol enrichment was achieved by incubating cells for 30 min with 5 mM MβCD + 0.5 mM cholesterol.

## Data availability

Cryo-EM electron density map of the two states human HCN3 (apo-HCN3 and cAMP-HCN3) has been deposited in the Electron Microscopy Data Bank: https://www.ebi.ac.uk/pdbe/emdb/(accession number EMD-35602 and EMD-35603), the fitted coordinate has been deposited in the Protein Data Bank, www.pdb.org (PDB ID code 8INZ and 8IO0). The cryo-EM data is shown in Table S1 for details. Further information and requests for resources and reagents should be directed to and will be fulfilled by the lead contact, J. Z. (zhangxiaokong@hotmail.com). Plasmids generated in this study are available from the lead contact upon request.

## Supporting information

This article contains supporting information.

## Conflict of interest

The authors declare that they have no conflicts of interest with the contents of this article.

## Figures and Tables

**Supporting Figure S7:**