# Supplementary material for: Cryo-EM structure of human HCN3 channel and its regulation by cAMP
Source: J Biol Chem. 2024 Apr 16;300(6):107288. doi: 10.1016/j.jbc.2024.107288 (PMC11126801; doi:10.1016/j.jbc.2024.107288)
Supplement: Supporting Figure Legends [file mmc1.docx]

**Supplementary Figure legends**

**Figure S1. The functional and biochemical characterization of human HCN3.**

**(A)** Voltage-dependent activation of HCN3 was assessed using whole-cell currents recorded from -30 to -140 mV for HCN3-wt with and without MBP tag.**(B)**Activation curves for HCN3-WTin the absence or presence of the MBP tag were generated from mean tail current activation curves, showing no clear difference between the two conditions.**(C)**Size-exclusion chromatography (SEC) was used to separate peak fractions of HCN3, which were used for cryo-EM analysis. **(D)**SDS-PAGE gel of peak fractions stained with Coomassie blue confirmed the predicted molecular weight of a single HCN3FL subunit, including the MBP tag (130 kDa). **(E)** The whole cell currents look like with and without the HCN3 constructs.

**Figure S2.Single-Particle Cryo-EM of the HCN3 Channel in the Ligand-Free State (Apo state).**

**(A)** Representative raw micrograph of the channel in the ligand-free state and the selected 2D class averages.**(B)** Flowchart of image processing for HCN3 particles.**(C)** The Gold standard Fourier Shell Correlation (FSC) curve of the final 3D reconstruction of HCN3, and the FSC curve for cross-validation between the map and the model of HCN3.**(D)** The density map of HCN3 colored by local resolution (Å).

**Figure S3. Single-Particle Cryo-EM of the HCN3 Channel in the cAMP-bound State.**

**(A)** Representative raw micrograph of the channel in the cAMP-bound state and selected 2D class averages.**(B)** Flowchart of image processing for HCN3 particles.**(C)** The Gold standard Fourier Shell Correlation (FSC) curve of the final 3D reconstruction of HCN3, and the FSC curve for cross-validation between the map and the model of HCN3.**(D)** The density map of HCN3 colored by local resolution (Å).

**Figure S4. Cryo-EM reconstruction density map of HCN3 in the apo state. Each channel subunit is color-coded.**

3D reconstruction of hHCN3-apo,with each subunit individually colored.Gray bars on

either side of the structure define the position (top and bottom) of the cell membrane.

**Figure S5. Sequence Alignment of HCN Channels.**

The alignment of HCN1-4 channels reveals conserved residues in the ion selectivity filter (located between the S5 and S6 helices). The residues involved in these functions are highlighted in a red box and are conserved across HCN channels. The secondary structures of the channels are depicted as ribbons for alpha helices and lines for loops.

**Figure S6. The half maximal inhibitory concentration (IC50) of cholesterol (left) and CHS(right).**

After removal of endogenous cholesterol by incubating cells in 5mM MβCD，different concentrations of cholesterol or CHS were applied with 5mM MβCD and whole-cell currents were recorded during -100mV pulse. Data points are mean±SEM of 3-5 cells.

**Figure S7.Pore of the HCN3 channel in two states.**

**(A)** The ion pore of the HCN3 channel is depicted with two subunits, viewed from within the membrane. The minimal radial distance from the center axis to the protein surface is indicated in gray.Selected residues facing the pore are in stick representation, and constricting residues are labeled. **(B)** The pore radius is shown in a plot color-coded as follows: apo (green) and cAMP-bound (blue).
