# Supplementary material for: Cryo-EM structure of human HCN3 channel and its regulation by cAMP
Source: J Biol Chem. 2024 Apr 16;300(6):107288. doi: 10.1016/j.jbc.2024.107288 (PMC11126801; doi:10.1016/j.jbc.2024.107288)
Supplement: Supporting Table S1 [file mmc2.docx]

**Table S1.**Cryo-EM data collection, refinement and validation statistics

|  | HCN3-apo  (EMD-35602)  (PDB 8INZ) | HCN3-cAMP  (EMD-35603)  (PDB 8I00) |
| --- | --- | --- |
| **Data collection and processing** |  |  |
| Magnification | 105,000 | 130,000 |
| Voltage (kV) | 300 | 300 |
| Electron exposure (e–/Å^2^) | 50 | 40 |
| Defocus range (μm) | -1.8-2.2 | -1.8-2.2 |
| Pixel size (Å) | 0.842 | 0.668 |
| Symmetry imposed | *C4* | *C4* |
| Initial particle images (#) | 1,347,234 | 1,990,846 |
| Final particle images (#) | 71,465 | 213,282 |
| Map resolution (Å) | 2.72 | 3.19 |
| **Refinement** |  |  |
| Initial model used (PDB code) | *de novo* | *de novo* |
| Model resolution (Å)  FSC threshold | 2.72  0.143 | 3.19  0.143 |
| Model composition  Non-hydrogen atoms  Protein residues  Ligands | 14920  1828  4 | 15052  1896  8 |
| r.m.s. deviations  Bond lengths (Å)  Bond angles (°) | 0.01  1.44 | 0.01  1.01 |
| Validation  MolProbity score | 0.99 | 1.43 |
| Ramachandran plot  Favored (%)  Allowed (%)  Disallowed (%) | 98.69  1.31  0.00 | 96.84  3.16  0.00 |

**References:**

1. Kucukelbir, A., Sigworth, F.J. & Tagare, H.D. Quantifying the local resolution of cryo-EM density maps. *Nat Methods***11**, 63-5 (2014).
